# LSD1 for the Targeted Regulation of Adipose Tissue

**DOI:** 10.3390/cimb45010012

**Published:** 2022-12-27

**Authors:** Li Chen, Xuan Sun, Daidi Chen, Qingjun Gui

**Affiliations:** Research Lab for Clinical & Translational Medicine, Hengyang Medical School, University of South China, Hengyang 421009, China

**Keywords:** LSD1, adipose tissue, obesity, epigenetics, metabolism

## Abstract

White and thermal (brown and beige) adipose tissue energy storage and oxidative regulation pathways play a central role in maintaining the energy balance throughout the body, and the dysregulation of these pathways is closely related to glucose and lipid metabolism disorders and adipose tissue dysfunction, including obesity, chronic inflammation, insulin resistance, mitochondrial dysfunction, and fibrosis. Recent epigenetic studies have identified the novel regulatory element LSD1, which controls the above parameters, and have provided new mechanistic possibilities for re-encoding the fate and function of adipocytes. In this review, we outline the current advances in adipocyte metabolism in physiology and disease and discuss possible strategies for LSD1 to alter the phenotype of adipose tissue and thus influence energy utilization to improve metabolic health.

## 1. Introduction

Long-term high calorie intake and lack of activity cause lipid metabolism disorders, which result in an energy imbalance, leading to obesity, diabetes, atherosclerosis, and a series of diseases—a serious threat to human health [1]. Adipose tissue is a key factor in the body, regulating lipid metabolism, which is equivalent to an energy converter in the human body. Brown and beige adipose tissue, regulated by their characteristic UCP-1, convert large amounts of triglycerides and glucose into heat energy and regulate body temperature, while white adipose tissue is mainly responsible for storing energy [2]. However, studies have shown that the impaired thermobiochemical function of these adipocytes is closely related to obesity [3,4]. Thus, understanding the functional mechanisms of brown and beige adipocytes and finding effective strategies to regulate their activities has aroused widespread scientific interest for improving metabolic health.

Epigenetic regulation is a mode of regulation that does not alter genetic DNA sequences but affects chromatin structures and gene expression [5]. Recently, it was found that epigenetic regulation is closely related to the occurrence and development of diseases such as atherosclerosis (As) [6,7]. Epigenetic regulation mainly includes DNA methylation, histone modification, microRNA regulation [8,9,10], in which histone modification is closely related to changes in the metabolic environment in the body [11]. Histone methylation is an important way of histone modification, including that at the arginine and lysine sites, and it is generally assumed that methylation will occur mainly at the N termini of histones H3 and H4 [12,13]. Lysine-specific demethylase (LSD1) has been found to play an important role in maintaining the metabolic properties of brown fat, and the specific knockout of LSD1 in brown adipocytes or its enzymatic activity in brown adipocytes triggers the whitening of brown adipose tissue [14]. LSD1demethyl—modified histone H3 lysine 4 (H3K4) inhibits the expression of related genes, finally inhibiting the activity of the Wnt signaling pathway, and then promotes the formation of brown adipose tissue. Scholars have also found that LSD1 regulates the metabolism of brown adipocytes in two ways. On the one hand, the binding of LSD1 to PRDM16 inhibits the expression of white fat-selective genes. On the other hand, LSD1 inhibition of glucocorticoid-activating enzymes occurs independent of PRDM16. Specific ablation of fat LSD1 decreases the mitochondrial fatty acid oxidation capacity of brown adipose tissue, reduces whole-body energy expenditure, and increases fat deposition [15]. In addition, LSD1 also affects the osteogenic differentiation of adipose-derived stem cells [16]. Based on the above, LSD1 plays a regulatory role in the epigenetics of adipose tissue.

## 2. Biological Function of the LSD1

In 1942, Conrad H. Waddington discovered that the expression of stably inherited genes during cell division and differentiation is not entirely dependent on DNA sequence, a phenomenon later called epigenetics [17]. Epigenetics, which has developed into a distinct discipline over the past few decades, refers to stably inherited phenotypic changes in gene or histone expression caused by chromatin modifications, but does not involve DNA sequence alterations. Epigenetics affects gene expression in a variety of ways, including DNA methylation, post-translational histone modification, and non-coding RNA regulation. These modifications and processing can individually or synergistically regulate gene expression, and also interact with the environment [18,19,20,21], thereby affecting a series of physiological activities of cells. The epigenetic model is crucial for activating or repressing the genes involved in cell proliferation, embryonic differentiation, signal transduction pathways, and dynamic balanced physiological processes and is a complex regulatory system [22,23]. At present, the research content of epigenetics is mainly focused on post-translational histone modification and DNA methylation. There are many ways to regulate post-translational histone modification, such as methylation, phosphorylation, acetylation, and ubiquitination. Among them, acetylation and methylation are the main histone modifications that regulate histone expression.

Lysine-specific demethylase 1 (LSD1) is a lysine-specific epigenetic modifier that selectively removes monomethyl and dimethyl-lysine from lysines 4 and 9 (H3K4 and H3K9, respectively) of methylated histone H3, thereby inhibiting or activating gene transcription programs. When LSD1 interacts with COREST, HDAC1/2, BHC80, and other transcriptional repressors or other transcriptional repressor complexes, LSD1 demethylates histone H3K4me1/2 and represses gene transcription [24,25]. However, when LSD1 interacts with nuclear hormones such as AR (androgen receptor) and ESR1 (estrogen receptor 1) in a ligand-dependent manner, the methylated H3K9me1/2 histone substrate is demethylated and plays a role in promoting the transcription of related genes [26,27,28]. In addition, several cancer-related studies have shown that LSD1 can also demethylase non-histone proteins, such as forkhead box A1 (FOXA1), hypoxia-inducible factor 1-α (HIF-1α), P53, DNA methyltransferase 1 (DNMT1), transcription factor E2F1, and tumor suppressor gene PTEN [29,30]. In recent years, the functions of LSD1 in various biological processes have been extensively studied, including its effects on cell cycle, cell differentiation, cell proliferation, stem cell self-renewal, and epithelial–mesenchymal transition [26,27,28,31]. Meanwhile, abnormal expression of LSD1 has been confirmed to lead to a variety of pathological states such as atherosclerosis, cancer, and virus-related diseases [32,33,34,35], suggesting that LSD1 can be used as a potential target for the treatment of these diseases.

## 3. Phenotype and Metabolism of Adipose Tissue

### 3.1. Types of Adipose Tissue

Adipose tissue is a highly dynamic organ distributed throughout the body and can be classified according to the cellular composition of different sites and its unique anatomical localization [36]. Different adipose tissues of the human body can be defined according to their location, size, cellular composition, and function and are mainly divided into white, brown, and beige adipose tissue [37]. White adipose tissue (WAT) constitutes the largest proportion of adipose tissue in the body, seen in major organs and perivascular, abdominal, and subcutaneous sites. White adipose tissue, once thought to be morphologically and functionally unspecific, is now considered dynamic, plastic, and heterogeneous and is involved in a wide range of biological processes, including energy balance, glucose and lipid utilization, blood pressure control, and host defense [38,39,40]. WAT stores excess energy in the form of triglycerides, and the increased accumulation of WAT, particularly in visceral depots, is a key determinant of cardiometabolic disorders, hypertension, and the relative risk of CVD12–17 [41]. Beige and brown adipose tissue account for less than 5% of adult adipose tissue, but they play a key role in lipid metabolism, glucose metabolism, and body temperature maintenance [42]. Brown adipose tissue is mainly stored in the interscapular region of infants and adults. Beige adipose tissue is a kind of white adipose tissue with scattered brown adipocytes, which has become of concern in recent years [43,44,45]. The massive expansion and phenotypic remodeling of adipose tissue during obesity have differential effects on specific adipose tissue stocks, and the relationship between the type of adipose tissue and its effect on cardiovascular function becomes particularly evident in the context of the heterogeneous phenotype of pericardiovascular adipose tissue. The increase in white adipose tissue greatly contributes to vascular dysfunction and cardiovascular disease [46,47,48,49]. Thermogenic brown and beige adipose tissue are associated with more positive cardiovascular effects than white adipose tissue.

**Figure 1:**
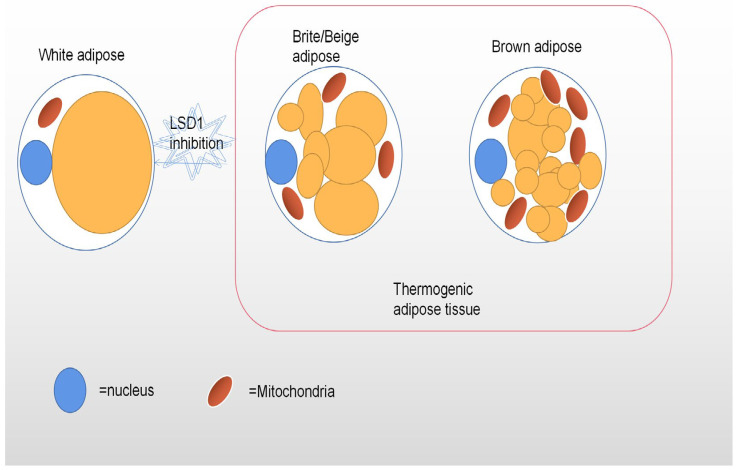
This figure depicts the different shapes of white, beige, and brown adipocytes. Brown and beige adipocytes have a high density of mitochondria and multiple lipid droplets, while white adipocytes have only one large lipid droplet and few mitochondria. Inhibition of LSD1 expression leads to the whitening of brown adipose tissue.

Adipose tissue (AT) is a highly heterogeneous endocrine organ composed of mature adipocytes, preadipocytes, fibroblasts, endothelial cells, and a series of inflammatory leukocytes. The heterogeneity among adipose tissues from different anatomical sites results from their inherent differences in cellular and physiological properties, which are caused by developmental origin, adipogenic and proliferative capacity, glucose and lipid metabolism, insulin sensitivity, hormonal control, thermogenesis, and angiogenesis [50,51,52]. Other factors that influence adipose tissue heterogeneity are genetic predisposition, sex, environment, and age. In the case of hyperplastic expansion of adipose tissue, these specific differences in adipose tissue translate into specific patterns of fat distribution and fat phenotypes, which are closely related to the risk of multiple metabolic diseases. The main functional cell type of AT is adipocytes. There are also obvious differences in the adipocyte morphology and function of different adipose tissue phenotypes. The main function of WAT is to promote a dynamic energy balance through the storage and release of fat (e.g., triglycerides) in response to nutrient intake and metabolic needs. Thus, white adipocytes contain a single large lipid droplet (monocular) and only a small amount of mitochondria [53]. At normal temperatures, beige adipocytes have more white phenotypes, with large lipid droplets and a lower expression of thermogenic genes, but the cold response, β-adrenergic stimulation, or motor activation leads to a robust upregulation of the thermogenic program, a process often referred to as “browning” [54]. Interestingly, since the epigenomic plasticity of beige adipocytes is temperature-dependent, there is also the ability to “whiten” in warm environments [55]. Unlike white adipocytes, heat-producing adipocytes (brown and beige adipocytes), especially brown adipocytes, have multicompartment lipid droplets and contain a large number of dense mitochondrial inner membrane uncoupled protein-1 (UCP1) mitochondria (Figure 1), which can oxidize fatty acid at a very fast rate [56]. Their highly active cellular metabolism is the result of unique protein mechanisms that allow the thermogenesis of adipocytes to participate in the futile recycling of normally stored metabolites. White and thermogenous brown/beige adipocytes come from different precursors, but development of both brown and beige adipocytes depends on the transcriptional coregulatory protein PR domain 16 (PRDM16) [57]. The thermogenic oxidation machinery of brown and beige adipocytes is regulated by transcription factors and cofactors, such as PR domain 16 (PRDM16) and the peroxisome proliferator-activating receptor γ coactivator-1 α (PGC-1 α), whose transcription factors bound to DNA cooperate to induce mitochondrial biogenesis and fatty acid oxidation of requiring heat production [58,59].

### 3.2. Metabolic Function of Adipose Tissue

Adipose tissue is an important regulatory factor in maintaining the balance of energy and the nutrient metabolism [60,61,62]. The primary function of white adipose tissue is to store energy in the form of triacylglycerol. As thermogenic adipocytes, brown and beige adipocytes rapidly adapt to use whole-body fuel to drive thermogenesis. The results of a series of experiments show that, as one would expect, adipocytes are extremely adaptive to the environment and can coordinate the storage of the fuel and energy stored by ineffective oxidation. The most representative lipothermogenesis mechanism is the regulation of mitochondrial inner membrane conductance through uncoupling protein 1 (UCP1), a mitochondrial inner membrane protein that consumes proton gradients across mitochondrial lipid bilayers. The result is that adipose tissue’s mitochondrial membrane potential can no longer be used for ATP synthesis, converting substrate oxidation energy into thermal energy. This uncoupling alleviates respiratory depression, which is normally caused by an excessive ATP/adenosine diphosphate (ADP) ratio in most cells [63]. The subsequent effect is a significant increase in the rate of respiration, substrate oxidation, and brown adipose tissue-mediated heat release. Another recently emerging mode of thermogenesis regulation in adipose tissue affects adipocyte metabolism by altering the REDOX state of cells. Genetic informatics or pharmacology elevated levels of mitochondrial reactive oxides (ROS) in adipocytes or the resulting increased oxidation of cellular thiols (the primary sensitive target of ROS) can lead to increased thermogenesis in brown adipose tissue [64,65]. At the same time, the systemic signals produced by the body, such as norepinephrine released from the sympathetic nervous system (SNS), play a significant role in regulating lipolysis and thermogenesis. In addition, beige adipocytes can convert to energy-storing white adipocytes within a few days of cessation of external stimuli, such as cold stimuli. In contrast, the circulating fatty acids produced through WAT lipolysis appear to be taken up by brown adipocytes via CD36 and subsequently used as fuel by their mitochondria and activated UCP1 adipose tissue, while being an active endocrine organ that secretes a variety of hormones (including estrogen in white adipose tissue) and inflammatory mediators that have either positive or negative effects in many obesity-related diseases. The maladaptation of adipose tissue is closely related to the occurrence and development of metabolic disorders, including insulin resistance, dyslipidemia, atherosclerosis, hepatic steatosis, and type 2 diabetes [66]. Chronic inflammation in white adipose tissue is largely caused by excessive caloric intake, which affects internal metabolism, leading to insulin resistance and subsequent adipocyte dysfunction [67]. Meanwhile, brown adipose tissue has a certain anti-obesity and antidiabetic effect.

Brown, beige, and white adipocytes respond to whole-body signaling, including SNS-driven stimuli, that rapidly alter fuel uptake and consumption and the production of various metabolite classes. In many cases, dramatic changes in adipose tissue-associated metabolite flow are a critical upstream regulator of adipocyte function. Studies have demonstrated that adipocyte-specific overexpression of Notch1 impairs thermogenesis and insulin sensitivity, leading to classical BAT whitening, and that pharmacological inhibition of Notch1 causes WAT browning and ameliorates high-fat diet-induced obesity [68,69]. We found that these alterations are all closely related to LSD1. As discussed in detail below, LSD1 has a profound effect on the phenotype and metabolite flow of adipocytes. It is noteworthy that regulating LSD1 expression affects the expression of certain metabolites and sufficiently changes the functional properties of adipocytes.

## 4. Possible Mechanisms by Which LSD1 Regulates Adipose Tissue

Recently, epigenetic changes were found to cause changes in genetic phenotypes, and the promoter sequence methylation level of the PRDM16 gene is closely related to the occurrence of obesity [70]. Additionally, LSD1 is a key part of regulating methylation, so we speculate that LSD1 may play an indispensable role in regulating the phenotype and metabolic process of adipose tissue. Further studies have shown that LSD1 maintains the characteristics of BAT through a dual action [14,53]. It cooperates with the transcription factor Nrf1 to positively regulate the expression of BAT-selected genes, and to actively repress the expression of WAT-selected genes through the recruitment of a core complex. Meanwhile, LSD1 affects the biological changes of adipose tissue by affecting the transcription of other biological factors involved in adipose differentiation, such as calcitonin. The BAT whitening observed upon depletion or inhibition of LSD1 in BAT is accompanied by a shift in the mode of adipocyte metabolism from oxidative to glycolytic metabolism, which is associated with the accumulation of diacylglycerides and triacylglycerides. This breakdown in BAT function, while accelerating the rate of weight gain, also improves glucose tolerance. In WAT, however, LSD1 is induced by cold and is involved in mediating the role of β3-adrenergic signaling, and adipose tissue gradually moves toward a thermogenic brown adipose tissue phenotype. Notably, it has recently been found that increasing LSD1 expression in mouse WAT promotes the development of beige adipocytes with thermogenic activity, browning WAT, and inhibiting the development of metabolic disorders such as obesity and type 2 diabetes in the body [71]. In addition, in mature white fat cells, the LSD1 expression level rises enough to activate mitochondrial biological thermogenesis (e.g., oxidative phosphorylation) and adjust the way of energy production and the fatty acid catabolism—such as the tricarboxylic acid (TCA) cycle, electron transfer chain (ETC), and β-oxidation—the pathways of gene expression in the knockout of LSD1, after significant changes. A deficiency of LSD1 in BAT not only leads to a decrease in its oxidative function, but also increases the glycolytic capacity [15,72]. Inhibition of LSD1 affects the metabolic remodeling of adipose tissue and leads to related defects, including insulin resistance, ectopic fat deposition, and chronic inflammation. These defects increase the risk of cardiovascular disease, diabetes, and non-alcoholic liver cirrhosis in obese patients and further aggravate the body’s metabolic disorders [73].

**Figure 2:**
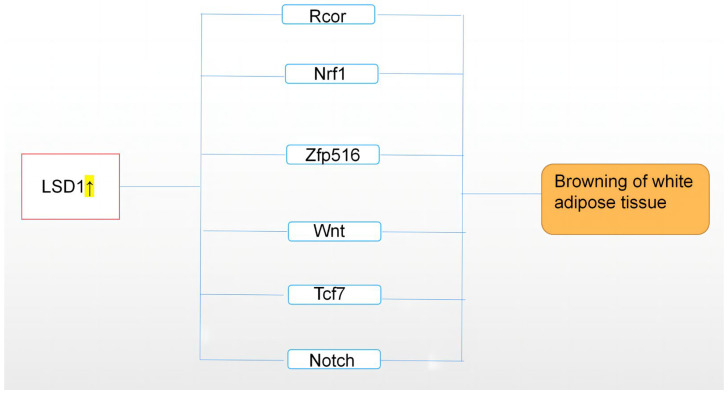
This figure describes the upregulation of LSD1 expression, which regulates adipose tissue through Rcor, Nrf1, Zfp516, Wnt, and other related pathways, resulting in browning of white adipose tissue, namely, “browning”. On the contrary, downregulation of LSD1 may lead to whitening of brown adipose tissue. The highlighted arrow indicates a sharp uptick in LSD1.

In summary, a series of data revealed LSD1 as a key regulator of WAT and BAT gene expression and metabolic functions. These findings provide a new regulatory pathway linking histone modifications and adipose tissue transformation to both mitochondrial oxidative capacity and whole-body energy balance. Below, we summarize the differentiation transition mechanisms of LSD1 in regulating BAT and WAT in recent years (Figure 2).

### 4.1. The Synergistic Inhibitory Effects of LSD1 and Rcor Jointly Regulate Adipose Tissue

#### Transformation

One theory is that LSD1 regulates adipose tissue transformation by coordinating it with the Rcor protein core complex, especially CoREST/REST corepressor 1 (Rcor1) and CoREST/REST corepressor 3 (Rcor3). LSD1 is demethylated together with the nucleosomal histone H3 lysine 4 (H3K4) residues, and acts in concert with the Corest/Rest corepressor 1 (Rcor1) to regulate cell fate through epigenetic repression of gene targets. All three Rcor proteins interact with the LSD1 and the erythroid megakaryocytic transcription factor-independent growth factor (GFI) 1b [74]. Yet, Rcor2 promotes LSD1-mediated nucleosome demethylation, as does Rcor1, while Rcor3 competitively inhibits this process. The addition of the SANT2 domain of Rcor1 to Rcor3 promotes the LSD1-mediated demethylation of the chimeric Rcor proteins. Gfi1b and LSD1 recruit Rcor3 to orthologous gene targets, leading to the repression of H3K4 demethylation in chromatin and the transcriptional derepression of these molecular mechanisms [75]. Notably, changes in the presence of the Rcor1/3 levels during erythroid differentiation and megakaryocyte differentiation further strengthen the antagonistic results. In BAT, the data suggest that LSD1 synergistically suppresses for the expression of WAT-selective genes in BAT by mutually regulating H3K4 and H3K9 methylation levels, in coordination with the core complex, especially Rcor1 and Rcor3 [76,77,78].

### 4.2. LSD1 Interacts with Nrf1 to Promote BAT Expression

In addition, the interaction of LSD1 with Nrf1 can maintain the thermogenic effect of adipose tissue. The LSD1 histone modifier requires a gene’s transcription start junction factor nuclear respiratory factor 1 (NRF1) and an active inducer, estrogen receptor-related α (ERRα), to transcriptionally activate common targets. The nuclear respiratory factor 1 (NRF1) transcription factor is considered a key factor in binding LSD1 to transcriptional start sites (TSS1). It has been previously reported that PGC-1α and NRF1, which are master regulators of mitochondrial biogenesis and metabolic adaptation, also upregulate the gene encoding Fundc1 during transcription and cooperatively regulate mitochondrial homeostasis. Fundc1 is a newly discovered mitophagy receptor protein, which can respond to cold stress in brown adipose tissue and promote thermogenesis in brown adipose tissue. NRF1 binds to the classical consensus site in the Fundc1 promoter, upregulates its expression, and enhances its phagocytosis by interacting with Lc3. Specific knockdown of Fundc1 in BAT results in reduced mitochondrial turnover and accumulation of functionally impaired mitochondria and inhibition of the PGC-1α–NRF1–Fundc1 pathway, leading to impaired adaptive thermogenesis in adipose tissue. Meanwhile, previous studies have shown that NRF1 associates with PGC-1 co-activator family members, promotes mitochondrial biogenesis and activity, and, together with LSD1, induces the expression of the genes involved in oxidative phosphorylation in white adipose tissue [79]. From the histone/protein modification process, LSD1 may function as a specific and universal cofactor for NRF1 [80]. Meanwhile, Duteil [81] found that LSD1 interacts with Nrf1 to activate the expression of BAT-selective genes to drive mitochondriogenesis and thermogenesis transcription in brown adipocytes, maintaining their BAT properties.

### 4.3. LSD1 Combined with Zfp516 and PRDM16 Regulates the Adipose Tissue Phenotype

On the contrary, it has been suggested that LSD1 regulates the phenotype of adipose tissue through direct interaction with Zfp516 and PRDM16. Zfp516 is a cold-induced transcription factor rich in brown fat (BAT) genes induced by cold stimulation and sympathetic stimulation through the cAMP-CREB/ATF2 pathway, and plays a key role in BAT gene program activation and thermogenesis [82]. Zfp516 binds directly to the proximal part of the UCP1 promoter, but not to the enhancer region that binds to other transcription factors, and interacts with PRDM16 to activate the UCP1 promoter. At the same time, Zfp516 directly interacts with LSD1, which promotes transcription of UCP1-rich and other BAT genes such as PGC1α to demethylates H3K9 in the BAT-rich thermogenic gene promoter region; thus, adipose tissue browning is not related to shivering thermogenesis [83,84]. Microarray sequence data have identified PRDM16 as a direct LSD1 anchor, and the expression of PRDM16 is reduced upon LSD1 knockdown in BAT. Recent studies have shown that LSD1 binds and interacts with PRDM16 to promote BAT transformation and to inhibit the expression of selected WAT genes [85]. However, co-immunoprecipitation, size-exclusion chromatography, and mass spectrometry have not provided direct evidence for the interaction of LSD1 and PRDM16 in BAT, and no clear results have shown that LSD1 directly acts upstream of PRDM16. Indeed, BAT from LSD1 KO mice using UCP1-CRE have shown reduced UCP1 expression, accompanied by an increase in H3K9 single-nucleotide and demethylation proximal to the UCP1 promoter, the site of LSD1 and Zfp516 binding. Interestingly, experimental work by some later individuals have identified the LSD1 complex containing Zfp516 and Prdm16 and Ctbp1/2, HDAC1/2, and others [86]. The complex has been found to repress WAT-specific genes by colocalizing in H3K4me1 demethylated regions. Thus, the more plausible speculation is that LSD1, Zfp516, and the PRDM16 complex act together to influence the adipose tissue phenotype.

### 4.4. LSD1 Regulates the Wnt Signaling Pathway and in Turn Affects Adipose Tissue

#### Differentiation

Although epigenetic mechanisms regulate the differentiation of multiple lineages, the epigenetic regulation of brown adipocytic differentiation can still be said to be poorly understood. The classical Wnt/β-catenin pathway is recognized as a key regulator of embryogenesis, tissue differentiation, and cell biological behavior and the dysregulation of this pathway leads to many diseases. A series of immunoprecipitation and gene analysis results have suggested that LSD1 may directly regulate Wnt signaling, while Wnt signaling is inhibited during brown fat differentiation. Meanwhile, expression profile and histone methylation profiling studies have shown that LSD1 inhibition causes increased H3K4me2 levels in the promoter region of Wnt pathway components, and increased expression of these genes activates Wnt/β-catenin signaling [87]. Thus, we speculate that LSD1 may promote brown adipogenesis by demethylating H3K4 in the promoter region of the Wnt signaling element and by inhibiting the Wnt pathway. However, the expression level of LSD1 does not change significantly during the brown adipocyte differentiation process, suggesting that its enrichment in Wnt pathway genes might be due to increased recruitment or retention. Further work is therefore needed to determine the molecular mechanisms by which LSD1 is enriched in these molecular mechanisms [88]. It has been noted that Wnt signaling inhibits general lipogenesis through both PPARG and C/EBPA. Furthermore, LSD1 has been reported to function in the early stage of white adipocyte differentiation by demethylating H3K9-2Me in the C/EBPA promoter region and by inhibiting the brown lipogenic transcriptional program by PGC1α [89], suggesting that LSD1 may still exert both white and brown fat-specific effects through the Wnt pathway.

### 4.5. LSD1 Mediates Tcf7-Related Factor Activation to Promote Brown Fat Differentiation

In fact, there is evidence that osteocalcin plays a vital role in the energy metabolism, establishing interesting communication between bones and other metabolic organs, including adipose tissue. Meanwhile, the Gprc6a gene, which encodes the G-protein-coupled receptor, acts as an osteocalcin receptor and its expression is activated by brown adipose differentiation. Moreover, osteocalcin can further enhance the expression of Gprc6a. Similarly, overexpression and knockout experiments have confirmed the key role of Gprc6a in osteocalcin-mediated thermogenic gene activation. Tcf7 and Wnt3a have been identified as the possible targets of osteocalcin signal transduction. Tcf/LEF family proteins form a complex with transcriptional coactivator β-catenin. T cell factor7 (Tcf7) belongs to the DNA-binding factor of the Tcf/LEF1 family, which is essential for the typical Wnt/β-catenin pathway [90]. However, Tcf7 regulates the activation of Gprc6a and UCP1 promoters independent of the Wnt/β-catenin pathway [91], so we speculate that the pathway regulated by Tcf7 may be independent of said Wnt/β-catenin pathway. In addition, PRDM16 and LSD1 have been shown to cooperate with Tcf7 in regulating the transcription of UCP1 and Gprc6a. Further studies have confirmed that the activity of Tcf7 may require thermogenic coactivator PRDM16 and histone demethylase LSD1 to play a role. Therefore, LSD1 may also affect the activation of Gprc6a and UCP1 promoters by regulating Tcf7, which leads to brown adipocyte differentiation [92].

### 4.6. Inhibition of NOTCH1 Expression by LSD1 “Brown” Albicized Adipose Tissue

Notch signaling is an important transduction pathway for intercellular communication, and the Notch receptor is a transmembrane protein containing an extracellular region (NEC), a transmembrane region (N ™), and an intracellular region (NIC) [93]. In mammals, with four Notch receptors (Notch 1, 2, 3, and 4) and five Notch ligands (Delta-like 1, 3, and 4 and Jaged 1 and 2). Starting from the Notch receptor binding to ligands on neighboring cells, a cascade of gene regulatory events occur to regulate cell proliferation, differentiation, apoptosis, and survival, thus determining cell fate. All of the receptors and ligands in the Notch signaling pathway are single-channel transmembrane proteins. It has been previously reported that the Notch signaling pathway plays a key role in regulating fat browning and energy balance. Inhibition of the Notch signaling pathway can promote beige adipogenesis and mitochondrial biosynthesis in porcine adipocytes lacking a functional UCP1 protein [94]. Inhibition of the Notch signaling pathway promotes the expression of specific genes and mitochondrial biosynthesis in beige adipocytes, and reduces the rate of obesity to some extent. A large number of studies have illustrated that LSD1 may inhibit NOTCH1 expression and downstream signaling by binding with the NOTCH1 gene [95]. This finding has been validated in the pathological progression of various cancers such as esophageal cancer and small cell lung cancer [96]. As mentioned earlier in this article, overexpression of NOTCH1 changes the whitening of adipose tissue. Then, it is possible that enhanced LSD1 expression may also improve obese and diabetic people by inhibiting a range of activities of NOTCH1 and subsequently leading to the transition of albicized adipose tissue to brown adipose tissue.

## 5. Conclusions

In summary, both BAT and WAT exhibit phenotypic plasticity, because each type can respond to temperature or hormonal stimuli, presenting corresponding morphological and functional characteristics. The distribution and proportion of white and brown adipose tissue have a certain degree of influence on body energy utilization, glucose and lipid metabolism, and mitochondrial oxidative phosphorylation and understanding the potential mechanisms of WAT and BAT development and promoting WAT “browning” may provide direction for combating and preventing obesity and related diseases. Increasing organismal BAT may be a treatment of current research interest for metabolic diseases, but the application of this idea is limited by the lack of a potential target or mechanistic understanding. Recently, the activity of LSD1 was found to be regulated by the body’s energy metabolism and cell signaling, suggesting that it may be a new sensor for regulating adipose tissue differentiation and metabolic function [97]. At the same time, a large number of research data have shown that LSD1 is a key factor regulating the gene expression and metabolic function of WAT and BAT. Upregulation of LSD1 expression can promote further browning of white adipose tissue, which will become a new hot spot in epigenetics. Although the regulation of the biological function of adipose tissue by LSD1 may also be affected by some factors such as endocrinology and immunology outside epigenetics. At the same time, some reasons for the conflicting results may be related to the differences in genetic models and the selection of experimental methods. However, it is undeniable that LSD1 is an important target for regulating the transformation of adipose tissue, and LSD1 can indeed become an important target for the treatment of obesity-related metabolic diseases, providing some new ideas for improving the pathophysiological state related to adipose tissue transformation, such as diabetes and atherosclerosis.

## Data Availability

Not applicable.
